# Music therapy integrated virtual reality education for preoperative anxiety in gynecological surgery

**DOI:** 10.1038/s41598-026-51676-8

**Published:** 2026-05-11

**Authors:** Yoo Seop Shin, Myung Sun Yeo, Sung Won Na, Jeong Min Kim, Soo Ji Kim

**Affiliations:** 1 Department of Anesthesiology and Pain Medicine, Yonsei University College of Medicine, Seoul, 03722 Republic of Korea; 2 Music Therapy Education, Graduate School of Education, Ewha Womans University, Seoul, 03760 Republic of Korea

**Keywords:** Virtual reality, Music intervention, Preoperative care, Anxiety, Gynecological surgery, Korean version of the Profile of Mood States, Diseases, Health care, Medical research, Psychology, Psychology

## Abstract

This study explored the impact of immersive virtual reality (VR) and music-guided breathing interventions on emotional states and satisfaction with educational content in female patients undergoing gynecologic surgery. A prospective randomized controlled trial was conducted with 140 female patients aged 20–65 years, assigned to four groups (*n* = 35/group): (1) written explanation only, (2) VR education with verbal breathing guidance, (3) VR education with neurologic music intervention and live breathing guidance, and (4) written explanation with neurologic music intervention. Psychological states were assessed using the Korean version of the Profile of Mood States. Satisfaction and comprehension were evaluated with a structured questionnaire. ANCOVA revealed a significant overall effect of group assignment on psychological responses (F = 3.693, *p* = .014). Group 3 reported lower mean anxiety and depression compared with Group 2, although targeted comparisons between these groups were not statistically significant. One-way analysis of variance revealed significant differences in satisfaction scores (F = 10.125, *p*<.001), with Group 3 showing higher satisfaction (M = 45.31, SD = 4.28) than Group 2 (M = 43.26, SD = 5.20). These findings suggest that VR-based preoperative education was associated with improvements in psychological outcomes and patient satisfaction. However, targeted comparisons between key intervention groups did not reveal statistically significant differences in anxiety and depression.

**Supplementary Information:**

The online version contains supplementary material available at 10.1038/s41598-026-51676-8.

## Introduction

Preoperative anxiety is a substantial concern among surgical patients, with reported incidence rates ranging from 11% to 80%^1^. Such anxiety can lead to pathophysiological responses, such as hypertension and tachycardia, thereby increasing perioperative morbidity^2^. Contributing factors include limited patient understanding of surgical procedures and anesthesia, as well as fear of postoperative pain^3^. Effectively addressing this anxiety is crucial, as unmanaged anxiety negatively impacts surgical outcomes and patient satisfaction.

Various educational interventions, such as written materials and digital platforms, have been developed to mitigate preoperative anxiety and have been shown to reduce anxiety levels while enhancing patient satisfaction^4^. Immersive technologies, such as virtual reality (VR), have emerged as practical tools to enhance patient understanding and alleviate anxiety through interactive experiences^5^. However, VR alone may be insufficient to fully address the emotional needs of patients, as it often lacks elements that promote relaxation and emotional comfort. Furthermore, excessively realistic VR content can sometimes increase anxiety owing to overstimulation or vivid imagery^6,7^.

To mitigate this issue, integrating therapeutic elements such as music into VR education has been proposed. Music therapy is known for its calming effects and has been effective in reducing preoperative anxiety in various surgical contexts, including gynecological procedures^8,9^. When combined with VR, music enhances the immersive experience and promotes physiological regulation, such as synchronization of heart rate and breathing. This effect is mediated through auditory-motor coupling and entrainment mechanisms, whereby rhythmic auditory stimuli guide respiratory cycles and promote autonomic stability^10,11^.

This study aimed to investigate the effectiveness of a music-integrated VR intervention in reducing preoperative anxiety and improving understanding of the surgical procedure and anesthesia among female patients undergoing gynecological surgery. This innovative multisensory approach represents a considerable advancement in patient-centered care, with the potential to substantially enhance the preoperative experience.

Accordingly, this study investigated whether integrating music-guided breathing into VR-based preoperative education improves psychological outcomes and patient experience. Specifically, (1) we examined changes in anxiety and depression states, and (2) differences in satisfaction and comprehension of educational content among intervention groups.

## Results

### Participant characteristics

**Fig. 1:**
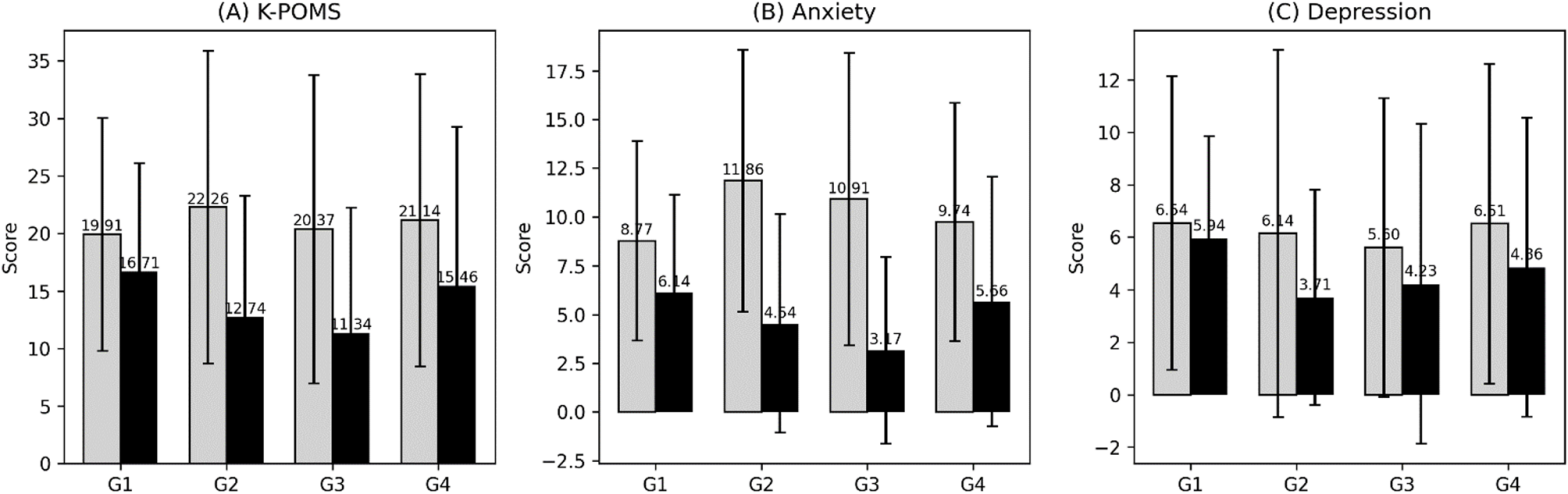
(**A**) K-POMS, (**B**) anxiety, and (**C**) depression scores in Groups 1–4. Bars represent mean pre- (light gray) and post-intervention (black) values, with error bars indicating standard deviations. Y-axis scales differ across panels to optimize visualization of each outcome.

**Fig. 2:**
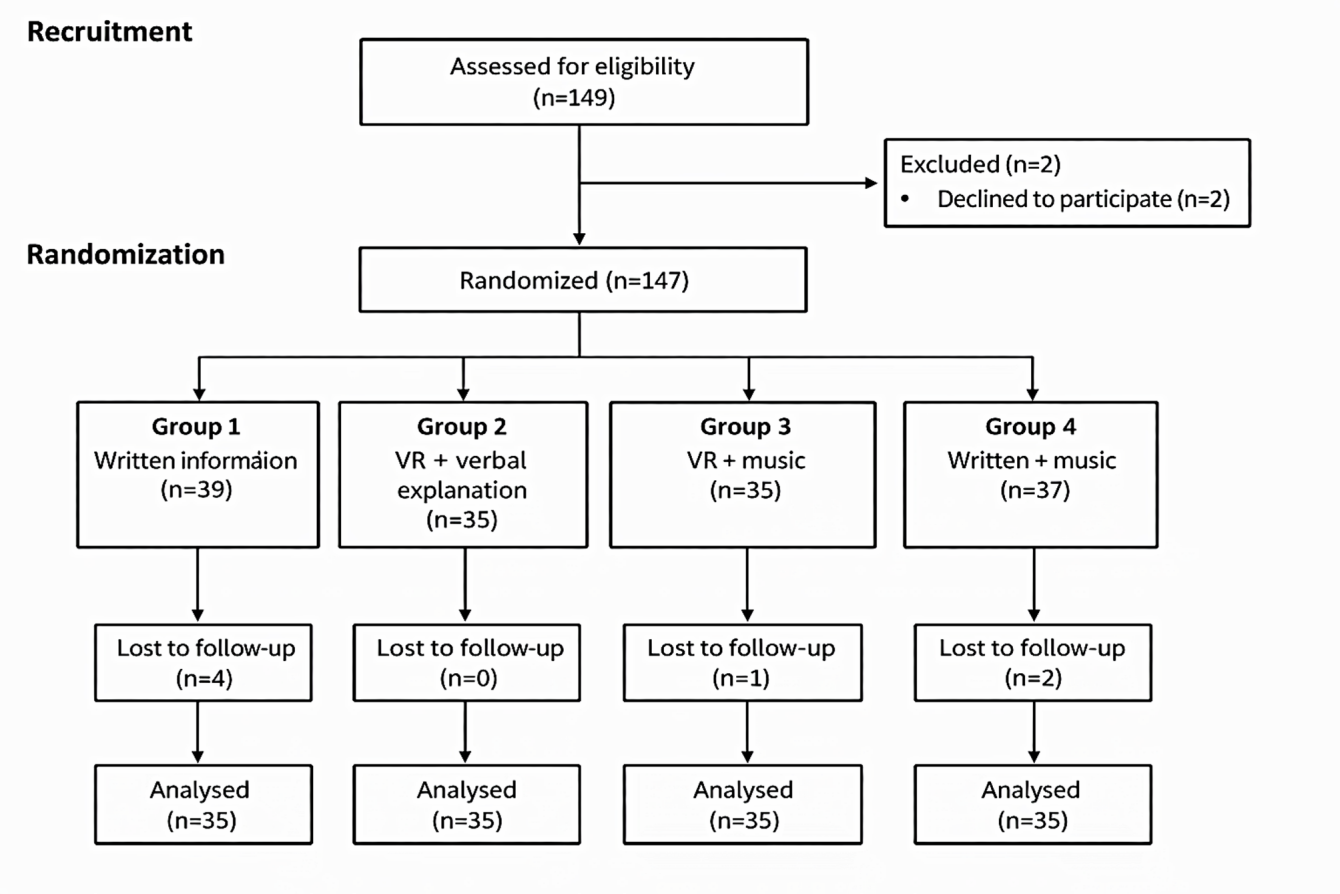
CONSORT flow diagram of participant recruitment, randomization, and analysis.

During the study period (March 2024–January 2025), a total of 140 patients were screened (Figs. 1 and 2). No harms or unintended events occurred in any group during the study.

Demographic characteristics—including age, surgery duration, and length of hospital stay—did not differ significantly among the groups (*p*>.05), supporting baseline equivalence and the validity of the results (Table 1).

**Table 1:** Demographic characteristics of the patients.

Variable	Group 1 (Written Only) *N* = 35	Group 2 (VR + Verbal) *N* = 35	Group 3 (VR + Music) *N* = 35	Group 4 (Written + Music) *N* = 35	t/χ²	*p*
Age *(M ± SD)*	42.71 ± 10.96	38.89 ± 7.73	40.14 ± 8.62	40.23 ± 8.30	0.336	0.581
Surgery duration (min)	158.37 ± 72.71	167.43 ± 92.02	157.14 ± 70.18	167.29 ± 71.40	0.585	0.627
Hospital stay (days)	1.94 ± 1.37	2.03 ± 2.08	2.08 ± 0.89	2.31 ± 1.61	0.481	0.696

VR, virtual reality.

### Psychological outcomes

The results showed that Groups 2 and 3 exhibited reductions in anxiety and depression scores on the post-test. Anxiety in Group 2 substantially decreased from M = 11.86 to M = 4.54, while anxiety in Group 3 declined from M = 10.91 to M = 3.17. For depression, Group 2 showed a reduction from M = 6.14 to M = 3.71, and Group 3 from M = 5.60 to M = 4.24 (Fig. 1). No significant between-group differences were observed in baseline measurements. ANCOVA indicated a significant effect of group assignment on psychological responses (F = 3.693, *p*=.014). Although the overall ANCOVA indicated a significant effect of group assignment, post-hoc comparisons between Groups 2 and 3 did not reveal statistically significant differences. Effect sizes were estimated as partial η² = 0.075 for total K-POMS, 0.079 for Anxiety & Fear, and 0.029 for Depression & Tension. However, focused comparisons between Groups 2 and 3 revealed no statistically significant differences (F = 0.043, *p*=.836), although Group 3 exhibited a lower mean negative mood state (Table 2).

**Table 2:** Psychological response (K-POMS) scores by Group (M ± SD).

Variable	Group 1 (Written Only) *n* = 35	Group 2 (VR + Verbal) *n* = 35	Group 3 (VR + Music) *n* = 35	Group 4 (Written + Music) *n* = 35	F	*p*	partial η²
Total K-POMS (Pre)	19.91 ± 10.06	22.26 ± 13.58	20.37 ± 13.38	21.14 ± 12.70	3.693	0.014*	0.075
Total K-POMS (Post)	16.71 ± 9.39	12.74 ± 10.5	11.34 ± 10.89	15.46 ± 13.76			
Anxiety & Fear (Pre)	8.77 ± 5.12	11.86 ± 6.68	10.91 ± 7.53	9.74 ± 6.1	3.870	0.011*	0.079
Anxiety & Fear (Post)	6.14 ± 5.04	4.54 ± 5.64	3.17 ± 4.8	5.66 ± 6.44			
Depression & Tension (Pre)	6.54 ± 5.6	6.14 ± 6.96	5.6 ± 5.74	6.51 ± 6.13	1.343	0.263	0.029
Depression & Tension (Post)	5.94 ± 3.93	3.71 ± 4.11	4.23 ± 6.14	4.86 ± 5.70			

Note. K-POMS = Korean version of the Profile of Mood States; VR = virtual reality; M = mean; SD = standard deviation.

Values are presented as mean ± standard deviation. Group differences were analyzed using one-way analysis of covariance (ANCOVA) controlling for age as a covariate. Effect sizes are reported as partial eta squared (η²).

### Satisfaction and comprehension outcomes

Regarding comprehension, Group 3 reported significantly higher overall satisfaction scores (M = 45.31) compared to Group 2 (M = 43.26; F = 19.035, *p*<.001) and demonstrated superior performance in the Information Delivery domain (M = 15.25 vs. M = 14.74; F = 4.664, *p*=.004).

In the Content Appropriateness domain, Group 3 exhibited significantly higher ratings (M = 18.62) than Group 2 (M = 17.89; F = 6.244, *p*=.001), particularly in the quality of evaluated materials (F = 63.075, *p*<.001). Similarly, in the Affective Response domain, Group 3 (M = 11.46) outperformed Group 2 (M = 10.56; F = 10.125, *p*<.001), with notable differences in interest, preference, and overall satisfaction. Significant group differences were observed in total satisfaction scores (F = 10.125, *p*<.001), with Group 3 (M = 45.31, SD = 4.23) attaining a higher mean than Group 2 (M = 43.26, SD = 5.19). Further item-level analyses revealed consistently higher ratings for Group 3 in comprehension, clarity, relevance, and overall satisfaction, with most differences reaching statistical significance (*p*<.05) (Table 3).

**Table 3:** Satisfaction with explanation scores by Group (M ± SD).

Domain	Variable	Group 1 *n* = 35	Group 2 *n* = 35	Group 3 *n* = 35	Group 4 *n* = 35	F	*p*-value	Effect size (partial η²)
Information delivery	Understanding	3.54 ± 0.56	3.80 ± 0.41	3.86 ± 0.36	3.51 ± 0.56	4.664	0.004*	0.093
	Clarity	3.14 ± 0.81	3.66 ± 0.54	3.74 ± 0.51	3.43 ± 0.56	6.668	< 0.001*	0.128
	Organization	3.17 ± 0.79	3.57 ± 0.50	3.77 ± 0.49	3.31 ± 0.68	6.372	< 0.001*	0.123
	Comprehensibility	3.37 ± 0.69	3.71 ± 0.46	3.86 ± 0.36	3.51 ± 0.56	5.709	0.001*	0.112
	Subtotal	13.32 ± 1.63	14.74 ± 1.29	15.25 ± 1.15	13.80 ± 1.33	4.664	0.004*	0.093
Content appropriateness	Need for material	2.57 ± 1.14	3.40 ± 0.88	3.57 ± 0.88	2.86 ± 0.94	8.076	< 0.001*	0.151
	Appropriateness	3.17 ± 0.71	3.60 ± 0.60	3.74 ± 0.44	3.34 ± 0.64	6.244	0.001*	0.121
	Content amount	3.26 ± 0.78	3.51 ± 0.61	3.63 ± 0.60	3.37 ± 0.65	2.102	0.103	0.044
	Material quality	1.37 ± 1.50	3.77 ± 0.43	3.89 ± 0.32	3.26 ± 0.70	6.308	< 0.001*	0.122
	Relevance	3.06 ± 0.91	3.66 ± 0.48	3.80 ± 0.41	3.31 ± 0.63	9.765	< 0.001*	0.177
	Subtotal	13.86 ± 2.90	17.89 ± 1.26	18.62 ± 1.05	16.20 ± 1.65	6.244	0.001*	0.121
Engagement and affective response	Interest	2.69 ± 1.25	3.51 ± 0.66	3.83 ± 0.38	3.31 ± 0.58	13.055	< 0.001*	0.224
	Preference	2.89 ± 1.02	3.54 ± 0.66	3.77 ± 0.43	3.31 ± 0.68	9.447	< 0.001*	0.172
	Satisfaction	3.00 ± 0.80	3.51 ± 0.70	3.86 ± 0.36	3.37 ± 0.69	10.125	< 0.001*	0.183
	Subtotal	8.83 ± 0.66	10.56 ± 0.88	11.46 ± 0.64	9.99 ± 0.72	10.125	< 0.001*	0.183
Overall satisfaction	Total satisfaction score	35.23 ± 7.64	43.26 ± 5.19	45.31 ± 4.23	39.91 ± 6.28	19.035	< 0.001*	0.296

Note. VR = virtual reality; M = mean; SD = standard deviation.

Values are presented as mean ± standard deviation. Group differences were analyzed using one-way analysis of variance (ANOVA). Effect sizes are reported as partial eta squared (η²).

## Discussion

Some limitations should be considered when interpreting the findings of this study. First, the sample was drawn from a single medical center, which may limit generalizability. Second, physiological measures of entrainment were not directly assessed. Third, the short-term design prevented evaluation of long-term outcomes. Fourth, information on additional factors potentially associated with preoperative anxiety, such as prior surgical experience, baseline psychological status, and medical or psychiatric history, was not systematically collected in this study. Future studies with multicenter samples and objective physiological measures are warranted.

This study investigated the efficacy of a music-integrated VR intervention in reducing preoperative anxiety and improving the patient experience among females undergoing gynecological surgery. The findings provide insights into the potential role of immersive VR technology, the added value of music, and factors that may influence psychological responses in this clinical context. Previous research has suggested that immersive VR interventions may help reduce preoperative anxiety and enhance patient preparedness by providing procedural familiarization and cognitive distraction^12–14^.

Although statistically significant differences in anxiety and depression between Groups 2 and 3 were not observed, the music-integrated intervention showed higher satisfaction and engagement scores. Group 3 demonstrated significantly higher scores for overall explanation satisfaction and comprehension, particularly in the domains of Information Delivery, Content Appropriateness, and Affective Response. The most notable difference was observed in the perceived quality of the educational material. These results suggest that, although the primary anxiolytic effect may partly reflect the immersive nature of VR combined with structured relaxation, the addition of music may further enhance the educational experience by promoting emotional engagement and facilitating information processing. Prior research has indicated that music interventions may support emotional regulation and cognitive engagement in medical settings, potentially improving patient satisfaction and perceived quality of care^15,16^. The use of water-based visual stimuli in the VR content may have contributed to the observed effects. Previous studies have suggested that natural water environments are associated with increased relaxation and reduced physiological stress responses, including lower blood pressure and heart rate^17^. In addition, pleasurable emotional experiences have been linked to activation in brain regions such as the orbitofrontal cortex, insula, and anterior cingulate cortex^18,19^. Visual processing of glossy or fluid-like surfaces, which are characteristic of water stimuli, has also been associated with activity in the visual cortex and its projections to the orbitofrontal cortex^20,21^. Although these neurophysiological mechanisms were not directly assessed in the present study, they may provide a plausible explanation for the enhanced emotional engagement and patient-reported outcomes observed with the VR-based intervention.

The music used in the breathing relaxation intervention was designed to align with a 4:6 respiration pattern and was tempo-matched to the average female breathing rate (111.87 bpm) to support rhythm-based entrainment. Rhythmic entrainment refers to the synchronization of internal physiological rhythms with external auditory stimuli and has been proposed as a possible mechanism through which music influences physiological regulation and emotional states^10,22^. However, the use of pre-recorded music and the limitations of the VR headset in enabling real-time therapist–patient interaction may have resulted in mismatches between the musical tempo and individual breathing rhythms. Although a 4-measure connecting section was included to allow patients to readjust their pace, individual differences in respiratory rate or intrinsic rhythmic perception (often referred to as the “inner beat”) may have limited full synchronization.

Although entrainment was not directly measured in this study, it may be hypothesized that stronger synchronization between music and physiological rhythms could enhance the effectiveness of relaxation interventions. Emerging research suggests that music may modulate autonomic nervous system activity and influence brain networks associated with emotional regulation and interoceptive awareness^23–25^. It has been hypothesized that synchronization between auditory stimuli and internal physiological rhythms may influence brain regions involved in emotional regulation, such as the insula and amygdala. Such neural mechanisms may contribute to emotional stabilization and reductions in anxiety.

This finding may highlight a potential limitation of fixed, pre-recorded audio in VR environments and underscores the possible advantages of incorporating adaptive technologies capable of real-time physiological monitoring. Systems that dynamically adjust musical tempo, rhythmic structure, or breathing guidance based on physiological signals may improve entrainment and potentially enhance therapeutic outcomes. Recent developments in biofeedback-driven digital health technologies suggest that integrating physiological monitoring with adaptive audiovisual interventions may increase intervention effectiveness and personalization^26^.

Nevertheless, despite limited real-time interaction, it may be hypothesized that elements such as pre-session guidance and rapport-building could have contributed to participants’ comfort and engagement. However, the establishment of a therapeutic alliance was not directly assessed in this study. Therapeutic alliance has been widely recognized as an important factor influencing treatment engagement and perceived support in psychosocial interventions^27^. The presence of supportive preparatory communication may therefore have contributed to participants’ comfort and engagement during the VR intervention.

Based on the findings and limitations of this preliminary study, several directions for future research may be considered. First, adaptive virtual systems capable of monitoring real-time physiological responses and dynamically adjusting music or breathing guidance could be developed to optimize and personalize entrainment effects. Second, longitudinal studies may be needed to examine the relationships between clinical and psychological variables and to incorporate these factors as covariates in multivariate analyses. Third, future research could evaluate the longer-term effects of VR- and music-based interventions on outcomes such as anxiety, depression, pain perception, and postoperative recovery. Finally, qualitative approaches, including in-depth interviews, may provide valuable insights into patients lived experiences and emotional responses to these interventions. Such insights may help inform the design of more responsive and patient-centered perioperative care strategies.

The findings of this study suggest that integrating music-guided breathing with VR-based preoperative education may enhance patients’ emotional engagement and satisfaction with educational content. This approach may represent a practical and non-invasive strategy for improving patient-centered perioperative care in gynecological surgery settings.

## Methods

This study was conducted and reported in accordance with the CONSORT guidelines for randomized controlled trials. Participants were first recruited before surgery. The primary and secondary outcomes were predefined before the initiation of the study.

Subsequently, outcome data were collected prospectively according to a predefined protocol.

### Procedure

**Fig. 3:**
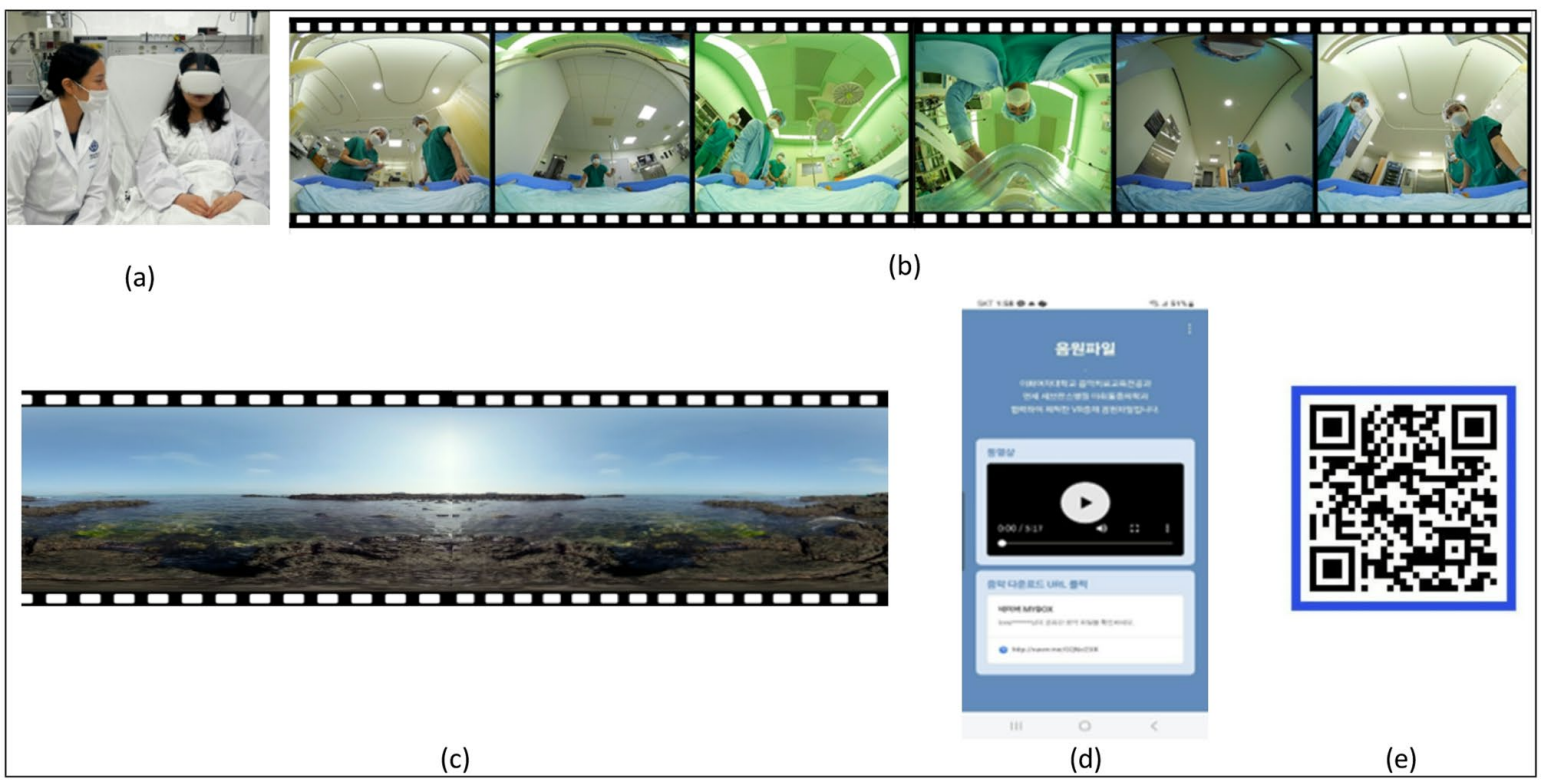
Details of the virtual reality (VR) intervention provided to Groups 2 and 3. The intervention video was presented as a continuous sequence: sections (**b**) and (**c**), followed by (**e**) and (**d**), to support self-directed training for patients. (**a**) A photo of a music therapist conducting the intervention. (**b**) The video clip played on the VR device shown in (**a**). The image features a series of stills from a simulation video illustrating the events on the day of surgery. It begins with verifying the name and registration number of the patient, followed by their transfer to the operating room, where they receive explanations about the procedure and anesthesia. Finally, in the recovery room, the patient is informed that the surgery was successful. A notable moment is captured in the fourth image, where the doctor begins administering anesthesia and says, “You may feel a bit of pressure as it goes in. You will fall asleep shortly,” while counting, “One, two, three…” (**c**) A VR relaxation scene used in both Group 2 and Group 3. (**d**) The start screen of a breathing relaxation music application designed for patient self-training on a mobile device. (**e**) A QR code providing access to the breathing relaxation music used in this study. All images in Fig. 3 were created by the authors or developed with appropriate permission.

**Fig. 4:**
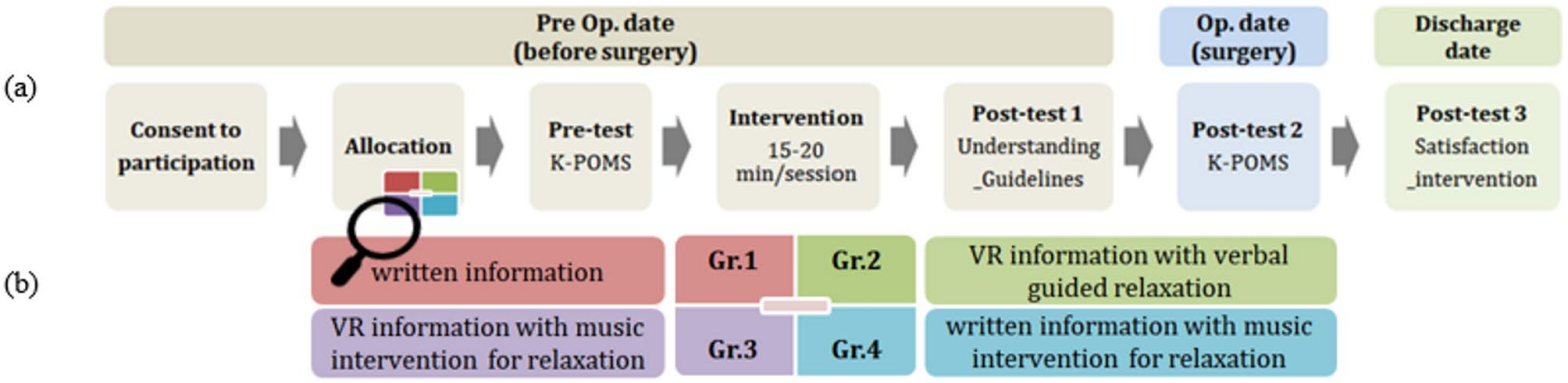
Overview of the study procedure and group allocation. (**a**) Presents the overall procedure of the study. (**b**) Enlarged view of the group allocation section from (**a**), illustrating the specific characteristics of each group.

This clinical trial was registered at ClinicalTrials.gov (NCT06728163) on December 11, 2024. Recruitment started before registration because of administrative delays. The study protocol, eligibility criteria, outcome measures, and statistical analysis plan were fully established prior to participant enrollment and were not modified thereafter. In this study, patients aged 20 to 65 years undergoing gynecological surgery were informed about the study and provided written informed consent by the attending gynecologist. They were categorized into four groups based on the type of educational material they received, along with a control group that received no intervention. After obtaining consent, patients were randomly assigned to one of the groups. One day before surgery, patients received a review of preoperative guidelines and completed a pre-test using the Korean version of the Profile of Mood States (K-POMS). Groups 1, 2, 3, and 4 received their respective interventions prior to surgery. On the morning of surgery, a post-test using the K-POMS was administered. Finally, on the day of discharge, the participants completed a satisfaction survey, marking the conclusion of the study (Figs. 3 and 4).

Interventions were delivered by board-certified anesthesiologists and professional music therapists. No additional eligibility restrictions for personnel were applied. Potential harms were defined as any adverse events related to the interventions, including dizziness, nausea, visual discomfort, or psychological distress during or after the VR or music-based sessions. Harms were assessed systematically by direct observation during the intervention and by asking participants to report any discomfort immediately after each session and at hospital discharge. No serious adverse events were reported during the trial. All methods were carried out in accordance with relevant guidelines and regulations. This study was conducted in accordance with the ethical principles of the Declaration of Helsinki. The study was approved by the Institutional Review Board (IRB) of Yonsei University College of Medicine (IRB No. 4–2023-1499 approved on January 5, 2024). Written consent was obtained from all participants prior to enrollment. Written informed consent was obtained from all individuals appearing in identifiable images for publication of these images in an online open-access format.

Hospitalized patients were screened based on inclusion and exclusion criteria, and a certified music therapist supervised all interventions in the ward. The attending obstetrician-gynecologist, research nurse, and music therapist visited patients preoperatively to explain the study and obtain written informed consent. Following consent, K-POMS was administered to assess preoperative anxiety. Participants were randomly assigned to intervention groups. The VR group viewed a surgical educational video (3 min) via Meta Quest 2, followed by a breathing relaxation exercise (4 min 32 s). The music group received brief instructions and performed inhalation–exhalation training synchronized with a pre-recorded breathing-relaxation music track.

### Rationale for sample size calculation subsection

The sample size for this study was calculated using the G*Power program, based on previous research related to this theme^28^. Assuming a statistical power of 0.95, four groups, and a Type I error rate of 0.05, the minimum required sample size for statistical significance was 112 patients. Considering a potential dropout rate of 20%, the final sample size was adjusted to 140 patients (rounded up from 116.66), with approximately 35 patients assigned to each of the four groups.

### Characteristics of the patients

A total of 140 patients, aged 20 to 65 years, participated in this study. All were classified as American Society of Anesthesiologists physical status I-III. Each was scheduled to undergo gynecological surgery under general anesthesia. This number includes two patients who dropped out and those whose surgeries were canceled. It also includes individuals facing cognitive, auditory, or visual impairments, or those who could not read the consent form due to illiteracy or non-proficiency in Korean, as stipulated by the participant selection criteria. All participants self-identified as Korean. Patients were enrolled between March 2024 and January 2025. Follow-up for the assessment of outcomes, including both benefits and harms, was conducted until January 2025.

Patients or the public were not involved in the design, conduct, reporting, or dissemination of this research.

Additional variables that may influence preoperative anxiety, such as previous surgical experience or psychological history, were not systematically collected in this study. Future studies may benefit from including these variables to better understand potential moderating factors.

### Treatment and intervention details by group

This study categorized gynecological patients undergoing general anesthesia into four groups based on the type of educational materials used: written materials only; VR materials accompanied by verbal relaxation interventions; VR materials combined with neurologic music intervention and live breathing guidance; and written materials paired with music relaxation intervention. Patients were randomly assigned to these groups to evaluate the effects of music-based relaxation on preoperative anxiety and depression, as well as to assess whether VR augments understanding of the procedure and improves overall satisfaction with the educational intervention.

### Rationale of research design

Previous research has reported varying effects on patient comprehension and satisfaction depending on the method of educational delivery, with some studies suggesting that VR may increase anxiety. Additionally, this study examined relaxation techniques that focused on breathing to alleviate anxiety, which were categorized as either verbal or music-based interventions. The participants were divided into four groups: (1) written education, (2) VR education with a verbal relaxation intervention, (3) VR education with a music-based relaxation intervention, and (4) written education combined with a music-based breathing relaxation intervention.

The specific procedures for each group are outlined below (Fig. 3).

#### 1) Group 1

Patients received written materials describing the surgical procedure.

#### 2) Group 2

Patients received education on the surgical procedure through VR videos viewed using a VR device (Meta Quest 2, Meta). The guidance was delivered via a pre-recorded narration by medical staff, mirroring the written information provided to Group 1. This was followed by a 2-minute breathing relaxation intervention featuring natural scenery and verbal guidance.

#### 3) Group 3

Patients viewed the same VR educational videos on surgical procedures as Group 2. In the latter part of the session, they also engaged in a music-based breathing relaxation intervention featuring the same video. The music, composed by two experts in neurologic music therapy, was synchronized with the inhalation–exhalation breathing pattern. The breathing rate was adjustable between 108 and 112 beats per minute (bpm), reflecting the average rate for females (111.87 bpm, Noh, 2020).

#### 4) Group 4

Patients received written materials along with a music-based breathing relaxation intervention.

**Fig. 5:**
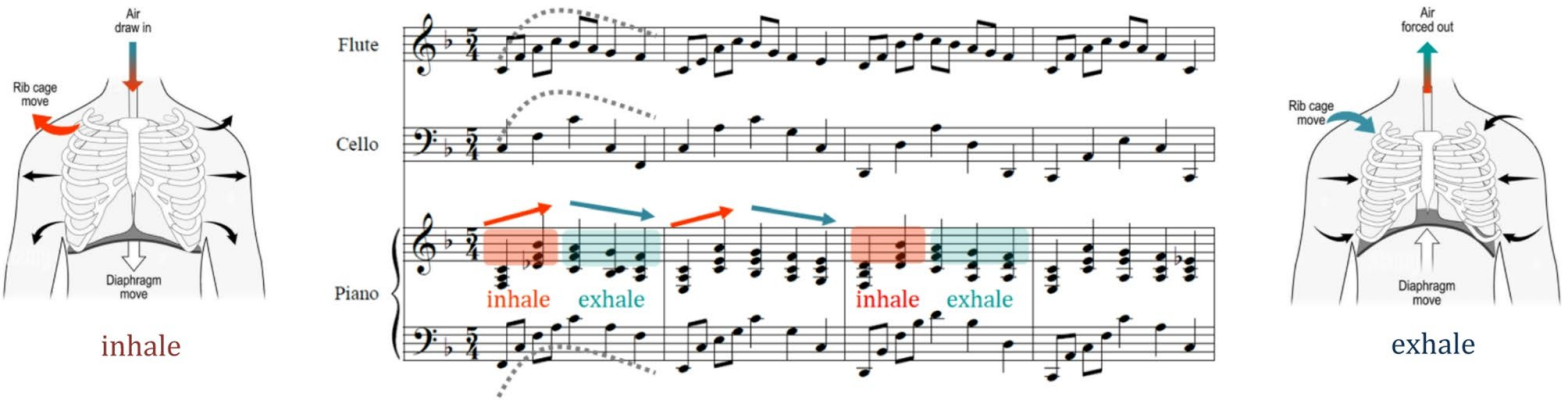
In standard music interventions, therapists observe patients and adjust music in real time. In this study, patients wore VR devices, making eye contact with the therapist difficult; pre-recorded music was used instead. This could cause mismatches between the music and patients’ performance. To address this, a 4-measure connecting section after 8 inhalation–exhalation cycles let patients adjust their pace. The music structure had 32 measures: eight in 5/4 time for the training section, repeated twice; a 2-measure rest in 3/4 time; a single 6/4 measure signaling the next breathing section; then eight measures of final training in 7/4 time, ending with one more 7/4 measure. Background music featured piano harmonies and flute and cello lines, which replaced the therapist’s verbal instructions.

The interventions and comparators were described in sufficient detail to allow replication. Written educational materials, VR content, and music-based breathing relaxation interventions were standardized across participants. Detailed descriptions of the music structure, breathing guidance, and VR environments are provided in the Methods and Figures (e.g., Figs. 3, 4 and 5). Additional materials, including the intervention manual, VR educational video, and music tracks, are available from the corresponding author upon reasonable request. No interim analyses were planned or conducted for this trial. No formal stopping guidelines were established, as the interventions were of short duration, non-invasive, and presented minimal risk to participants.

### Randomization: sequence generation

Simple randomization was employed using a computer-generated random number table with a 1:1:1:1 allocation ratio for the four groups. No stratification or blocking methods were applied, blinding of participants and personnel was not feasible due to the nature of the educational and music interventions. Outcome assessors were also not blinded, as the primary outcomes were patient-reported, and no restrictions were imposed on the random allocation sequence. The random allocation sequence was generated using a computer-based random number table in a statistical software program, with an allocation ratio of 1:1:1:1 for the four groups. The sequence was produced prior to participant enrollment and was not accessible to the investigators who conducted the interventions. The sequence was generated by the research team, and participants were assigned to groups after screening.

## Materials

Both written and VR educational materials consistently presented the standard preoperative guidelines established by the SMS (Kim, Lee, Kim, & Kim, 2016). Patients in Groups 2 and 3 used VR devices and headphones, which were pre-sterilized using a UV sterilizer designed for medical equipment, to view the customized educational content developed for this study. The educational video visually guided patients through the process of entering the operating room and lasted approximately 3 min, followed by a 2-minute breathing relaxation intervention. The VR video included continuous visual scenes of gentle water ripples throughout the session. The selection of water-based visual stimuli was informed by prior evidence suggesting that natural water environments are associated with positive affect and relaxation. This content was therefore incorporated to enhance the calming effect of the intervention. The VR educational content was developed by the authors in collaboration with Severance Hospital medical staff and commissioned external production partners. All images presented in Fig. 3 were either created for this study or developed under the authors’ supervision. Written informed consent was obtained from all individuals appearing in identifiable images. All materials are owned by or licensed to the authors and were used with appropriate permission.

### Rationale for music and intervention

The breathing relaxation intervention used in this study was developed based on examples from previous research cited in Yu and Song^29^. The instructions for the breathing technique were as follows: “Inhale slowly through your nose for 4 s, allowing your abdomen to expand outward. Then, exhale slowly through your mouth for 6 s while making a ‘hu’ sound, imagining your abdomen moving inward.”

Drawing from prior studies that established a 4:6 ratio for inhalation and exhalation as one cycle of diaphragmatic breathing^30^, this study recommended performing 10 cycles per set, with a total of three sets per day (Chang et al., 2007; Chang et al., 2009; Shim & Chang, 2005). This intervention was incorporated into both the verbal instructions and audio materials used in the music-based interventions. In the final set of three sessions, the duration of inhalation and exhalation was increased compared to the previous two sessions to deepen the practice. Additional harmonic progressions and instrumental timbres were incorporated, and the music was transposed to distinguish each set, thereby facilitating repetitive performance. The total duration of the music was 4 min and 32 s (Fig. 5).

All music-based interventions were led by the same certified music therapist with a master’s degree and over 8 years of clinical experience, ensuring procedural consistency. Intervention procedures were standardized before the study using protocols and training sessions. Treatment fidelity was ensured with scripts, fixed-duration materials, and therapist supervision.

### Measures

#### The K-POMS

Originally developed by McNair, Lorr, and Droppleman in 1971, the scale was adapted for Korean use by Kim et al. in 2003. The scale includes 65 items that assess six subscales: anxiety, depression, anger, vigor, fatigue, and confusion. The K-POMS has a content validity index of 2.66 out of 5 and a Cronbach’s α of 0.93 for all items, with individual subscale reliabilities ranging from 0.67 to 0.90, indicating strong internal consistency. Higher vigor scores indicate better outcomes, while lower scores on the negative emotional subscales also reflect better outcomes. Anxiety, fear, depression, and tension were assessed using a 5-point Likert scale ranging from 0 (not at all) to 4 (very much so).

#### Comprehension of explanation and educational satisfaction

This is a 12-item tool initially developed by Lim and Jeong in 1999 for web-based virtual classes and later adapted by Choi (2015) to access smartphone-based educational videos related to bowel preparation prior to colonoscopy. It evaluates aspects such as appropriateness, interest, and comprehension of the educational content, using a 4-point scale (1 = not at all to 4 = very much so) to indicate satisfaction levels. The tool demonstrated strong reliability, with Cronbach’s α of 0.84 at the time of development and 0.93 in Choi’s study. The total evaluation takes approximately 3 min to complete.

#### Statistical analysis

Data were analyzed using SPSS Statistics version 27. A one-way ANCOVA (Analysis of Covariance) assessed group differences in anxiety and depression, controlling for age as a covariate. A one-way analysis of variance was used to evaluate group differences in comprehension of explanation scores.

Planned pairwise comparisons were specified a priori based on the study objectives, with a particular focus on comparisons between the two VR groups, which differed primarily in the inclusion of music-guided breathing. To control for multiple testing, Bonferroni-adjusted post hoc comparisons were applied where appropriate. Analyses of subdomain outcomes were considered exploratory. All statistical tests were two-sided, and a p value < 0.05 was considered statistically significant.

## Conclusions

In summary, this preliminary study suggests that integrating music with VR-based preoperative education may enhance emotional engagement and subjective satisfaction among patients undergoing gynecologic surgery, even when measurable changes in anxiety and depression are limited. These findings indicate that incorporating multisensory and emotionally supportive elements into VR-based education may contribute to more patient-centered perioperative care and improve the overall patient experience. Future studies incorporating adaptive physiological feedback systems and longitudinal follow-up may further clarify the mechanisms underlying these effects and evaluate the potential long-term clinical benefits of music-integrated VR interventions.

## Supplementary Information

Below is the link to the electronic supplementary material.

Supplementary Material 1

Supplementary Material 2

## Data Availability

The datasets generated and/or analyzed during the present study are available from the corresponding author upon reasonable request. All shared datasets will be fully de-identified and provided together with a data dictionary. The statistical code used for the analyses will also be available upon request. In accordance with institutional and ethical regulations, access will be restricted to academic, non-commercial purposes and will be provided via secure institutional channels. Owing to ethical considerations and patient confidentiality, access to the data will be restricted to de-identified and limited portions deemed appropriate for academic use.
