# Supplementary material for: Music therapy integrated virtual reality education for preoperative anxiety in gynecological surgery
Source: Sci Rep. 2026 May 11;16:21516. doi: 10.1038/s41598-026-51676-8 (PMC13351033; doi:10.1038/s41598-026-51676-8)

# Detailed Protocol Document

# 1. General Information

Protocol Title: Immersive Virtual Reality Music Therapy as a Preoperative Intervention to Alleviate Anxiety and Enhance Patient Experience in Gynecological Surgery
Protocol ID: NCT06728163
Date of Document: October 11, 2025
Principal Investigator: Jeong Min Kim, M.D., Ph.D. (Department of Anesthesiology and Pain Medicine, Yonsei University College of Medicine, Seoul, Korea)
Co-Investigators: Yoo Seop Shin, M.D.; Myung Sun Yeo, Ph.D.; Sung Won Na, M.D.; Soo Ji Kim, Ph.D.
Sponsor/Funding: Self-funded by authors (no external funding)

Trial Registration: ClinicalTrials.gov Identifier: NCT06728163, First submitted: October 22, 2024; First Posted: December 11, 2024; Last update posted: December 11, 2024.

# 2. Summary

This randomized controlled trial (RCT) investigated whether immersive virtual reality (VR) combined with music-guided breathing reduces preoperative anxiety and improves comprehension and satisfaction among female patients undergoing gynecological surgery. A total of 140 patients aged 20–65 years were randomized into four groups. Interventions included written education only, VR education with verbal breathing guidance, VR with neurologic music intervention, and written education with music intervention. Primary outcome: preoperative anxiety and depression (K-POMS). Secondary outcome: satisfaction and comprehension of preoperative education.

# 3. Background and Rationale

Preoperative anxiety is common in gynecological surgery, with potential adverse effects on perioperative outcomes. Conventional education methods (written/verbal) often fail to address emotional distress. VR has emerged as an effective tool for enhancing comprehension but may lack emotional comfort. Music therapy has established anxiolytic effects. This study integrates VR with music-guided breathing to optimize both comprehension and emotional well-being.

# 4. Objectives

Primary Objective: To evaluate the effect of VR combined with music-guided breathing on reducing preoperative anxiety and depression.

Secondary Objectives: To assess comprehension and satisfaction with preoperative education across different intervention groups.

# 5. Study Design

Design: Interventional (Clinical Trial), randomized allocation (1:1:1:1), open-label (no blinding), primary purpose: supportive care/educational intervention.
Setting: Yonsei University College of Medicine, Seoul, Korea.
Duration: March 2024 – January 2025.
Arms (4 groups):
1. Written explanation only.
2. VR education with verbal breathing guidance.
3. VR education with neurologic music intervention and live breathing guidance.
4. Written explanation with music intervention.

# 6. Study Population

A total of 140 participants were planned for recruitment, consistent with the sample size calculation and the registered trial record.

Inclusion Criteria:
- Female only, 20–65 years old
- ASA physical status I–III
- Scheduled for gynecological surgery under general anesthesia
- Able to provide informed consent

Exclusion Criteria:
- Cognitive, auditory, or visual impairment
- Illiteracy or inability to read Korean consent
- Cancellation of surgery

# 7. Interventions

Group 1: Written preoperative education only.
Group 2: VR preoperative education with 2-min verbal breathing relaxation.
Group 3: VR education with music-guided breathing (neurologic music composed for 4:6 breathing cycle, tempo 108–112 bpm).
Group 4: Written education plus music-guided breathing.
Standardization: All educational content based on institutional preoperative guidelines.

# 8. Study Assessments and Outcomes

Primary Outcome:
- Preoperative anxiety and depression measured using Korean version of Profile of Mood States (K-POMS).
K-POMS Anxiety/Depression [Time Frame: Baseline (pre-operative), Post-Operative Day 0 (operation day)]

K-POMS Anxiety/Depression: This scale calculates scores based on 65 items assessing six subfactors: anxiety, depression, anger, vitality, fatigue, and confusion. For the subfactors, higher scores for vitality are better, while lower scores for the negative psychological factors are better. In this study, 9 items related to anxiety and 15 items related to depression were measured using a 5-point Likert scale (4 points being "strongly agree" and 0 points being "strongly disagree").

Secondary Outcomes:
- Satisfaction and comprehension using validated 12-item questionnaire.

Understanding of the explanation and educational satisfaction: These are based on a satisfaction assessment tool for web-based virtual classes developed by Im Jeong-hoon and Jeong In-seong (1999), which was modified and improved by Choi Mi-hee (2015) to evaluate the satisfaction of smartphone-based colonoscopy bowel preparation video education. Out of the 12 items in the original tool, 7 items that can be evaluated simultaneously with a written tool were used. The original tool measures aspects such as the appropriateness of educational content, methods, design, material organization, interest, and understanding, with a total of 12 items. It uses a 4-point scale (1: Not at all, 4: Very much), where higher scores indicate higher satisfaction. The reliability of the tool at the time of development was Cronbach's α = 0.84, and in Choi Mi-hee's study (2015), the reliability was Cronbach's α = 0.93. The evaluation takes approximately 3 minutes.

Timing of Assessments:
- Pre-test (1 day before surgery)
- Post-test (day of surgery, morning)
- Satisfaction survey (day of discharge)

# 9. Methodology

Randomization: Computer-generated random number table (1:1:1:1 allocation).
Blinding: Not feasible for participants or personnel due to intervention nature; outcome assessors not blinded.
Data Collection: Standardized questionnaires administered by trained staff.

# 10. Statistical Considerations

Sample Size Calculation: G*Power, power=0.95, α=0.05, four groups → minimum 112; adjusted to 140 considering 20% dropout.
Analysis: ANCOVA for K-POMS, ANOVA for satisfaction, Bonferroni correction for post-hoc tests.
Software: SPSS v27.

# 11. Ethical Considerations

Ethics Approval: Institutional Review Board, Yonsei University College of Medicine (IRB No. 4-2023-1499).
Consent: Written informed consent obtained from all participants.
Safety Monitoring: Participants observed for dizziness, nausea, visual discomfort, psychological distress. No adverse events reported.

All records will be retained for 3 years after study completion, with access restricted to the PI and designated staff only.

# 12. Monitoring and Safety

Minimal risk intervention.
No interim analyses planned.
Monitoring performed during and after sessions by attending anesthesiologists and music therapists.

Data Monitoring: No Data Monitoring Committee was established due to the minimal-risk nature of the intervention. Responsible party: Jeong Min Kim, M.D., Ph.D., Yonsei University College of Medicine.

# 13. Expected Outcomes

Hypothesis: VR with music-guided breathing will yield greater reductions in preoperative anxiety and improved satisfaction compared to VR or written education alone.

# 14. Dissemination and Publication

Results will be published in peer-reviewed journals and presented at academic meetings.
Data sharing will follow institutional and ethical guidelines (de-identified datasets available upon request).

# 15. References

1. Abate, S.M., Cheko, Y.A., & Basu, B. (2020). Global prevalence and determinants of preoperative anxiety among surgical patients: a systematic review and meta-analysis. *International Journal of Surgery Open, 25*, 6–16
2. Burrai, F., Ortu, S., Marinucci, M., De Marinis, M.G., & Piredda, M. (2023). Effectiveness of Immersive Virtual Reality in People with Cancer Undergoing Antiblastic Therapy: A Randomized Controlled Trial. In Seminars in Oncology Nursing 39(4).
3. Chen, H., Mo, L., Hu, H., Ou, Y., & Luo, J. (2021). Risk factors of post operative delirium after cardiac surgery: 13
4. A meta-analysis. *Journal of Cardiothoracic Surgery, 16*(1), 113.
5. Choi, M. H. & Song J. A. (2017). Effects of video assisted education using smartphone on bowel preparation for colonoscopy, *Journal ofKorean Academy andFundamentalNursing, 24*(1), 60-71.
6. Eijlers, R., Utens, E. M., Staals, L. M., de Nijs, P. F., Berghmans, J. M., Wijnen, R. M., ...& Legerstee, J. S. (2019). Meta-analysis: systematic review and meta-analysis of virtual reality in pediatrics: effects on pain and anxiety. *Anesthesia and analgesia, 129*(5), 1344.
7. Freedman, N.S., Kotzer, N., & Schwab, R. J. (1999). Patient perception ofsleep quality and etiology of sleep disruption in the intensive care unit. *American Journal of Respiratory andCritical Care Medicine, 159*(4), 1155-1162.
8. Friedrich, S., Reis, S., Meybohm, P., &Kranke, P. (2022). Preoperative anxiety. *Current Opinion in Anaesthesiology, 35*(6), 674-678.
9. Kim, K. Y., Seo, H. J., Min, S. S., Park, M., & Seol, G. H. (2014). The Effectof 1, 8‐Cineole Inhalation on Preoperative Anxiety: A Randomized Clinical Trial. Evidence‐Based Complementary and Alternative Medicine, 2014(1),820126.
10. Kim, D. J., & Chun, S. J. (2014). Design and application of a virtual reality-basedteaching model forfield based learning activities. *Journal of The Korean Association of Information Education,18*(1), 133-142.
11. Kim, E. J., Lee, S. I., Jeong, D. U., Shin, M. S., & Yoon, I. Y. (2003). Standardization and reliability and validity of the Korean edition of profile of mood states(K-POMS). *Sleep Medicine and Psychophysiology*, 10(1): 39-51.
12. Kim, J. H., Lee, E. H., Kim, S. R., & Kim, S. R. (2016). Factors affecting discharge delay in lumbar spinal surgery patients who were treated according to a critical pathway*.Korean Journal of Adult Nursing, 28*(1), 43-52.
13. Lee, E. N. Cho, J., Kim, M., Lee, E.,Lee, Y., Choi, E., & Lee. H. (2012). Effects of sleep promotion intervention on sleep quality and occurrence of delirium in intensive care unit patients. *Journal of Critical Care Nursing, 5*(1), 23-33.
14. McNair, DM, Lorr, M and Droppelman, L (1971).*Manual: Profile of Mood States*. San Diego, CA: Educational and Industrial Testing Service.
15. Roh, S. G. (2020).Analysis of cardiopulmonary resuscitation during mainstretcher transport. *Korean Journal of Emergency Medicine Service, 24*(2), 39-50.
16. Sengkeh, M. Y., & Chayati, N. (2021). Audiovisual virtual reality distraction in reduction of pain and anxiety intention in post-operative patients: A review study. *Open Access Macedonian Journal of Medical Sciences, 9*(F), 76-80.
17. Singh Solorzano, C., Steptoe, A., Leigh, E., Kidd, T., Jahangiri, M., & Poole, L. (2019). Pre-surgical caregiver burden and anxiety are associated with post-surgery cortisol over the day in caregivers of coronary artery bypass graft surgery patients*. International Journal of Behavioral Medicine, 26*(3), 316–322.
18. Tan, D. J. A., Polascik, B. A., Kee, H. M., Hui Lee, A. C., Sultana, R., Kwan, M et al. (2020). The Effect of perioperative music listening on patient satisfaction, anxiety, and depression: a quasi experimentalstudy. *Anesthesiology Research and Practice*, 1-6. 14
19. Weingarten, S. J., Levy, A. T., & Berghella,V. (2021). The effect of music on anxiety in women undergoing cesarean delivery: a systematic review and meta-analysis. *American Journal of Obstetrics & GynecologyMFM*, *3*(5), 100435.
20. Zemła, A.,Nowicka-Sauer, K., Jarmoszewicz, K., Wera, K., Batkiewicz, S., & Pietrzykowska, M. (2019). Measures of preoperativeanxiety. *Anaesthesiology Intensive Therapy, 51*(1), 66-72.

**16. Annexes (14)**

The annex includes questionnaires (K-POMS, Satisfaction tool), sample consent forms, and educational materials (written scripts, VR screenshots, music scores).
**- Questionnaires (K-POMS, Satisfaction tool)**

#
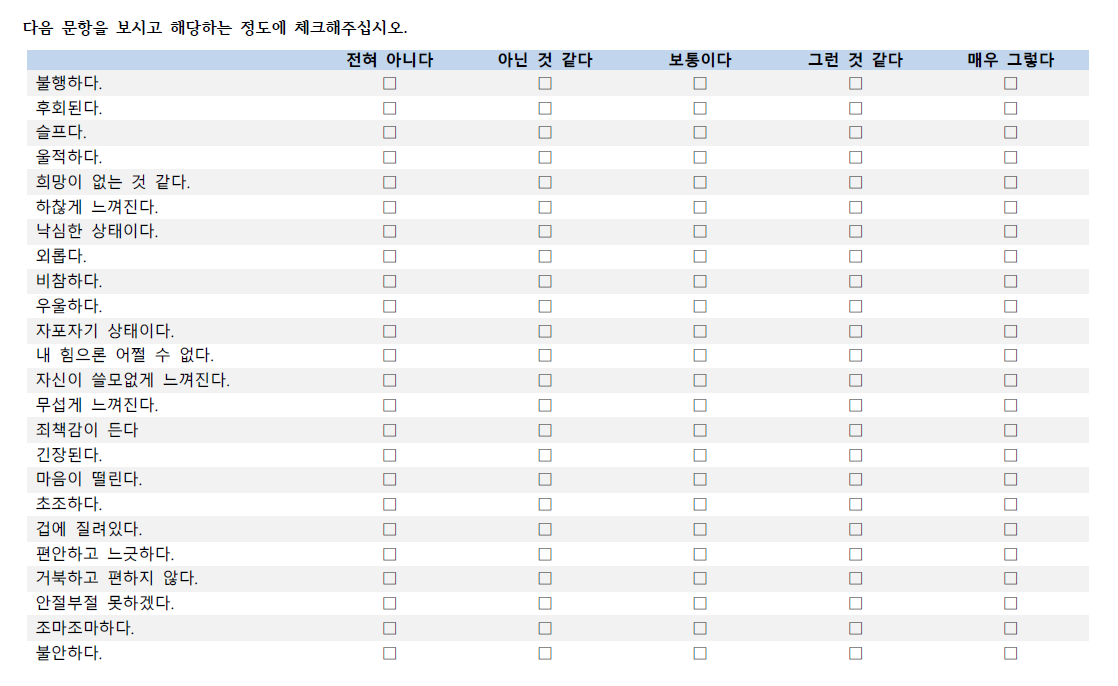


#
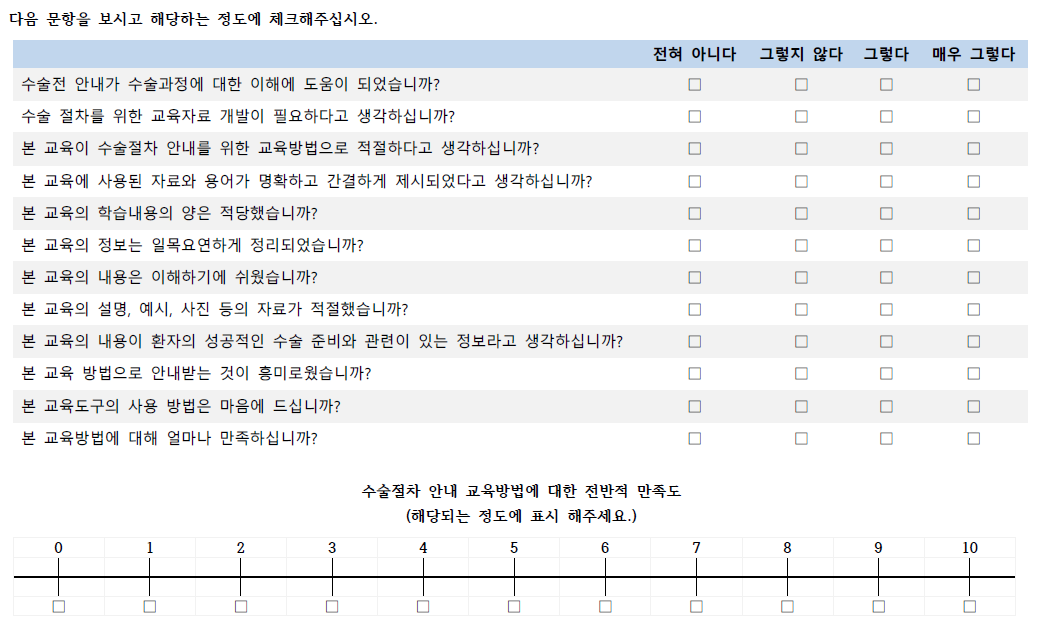

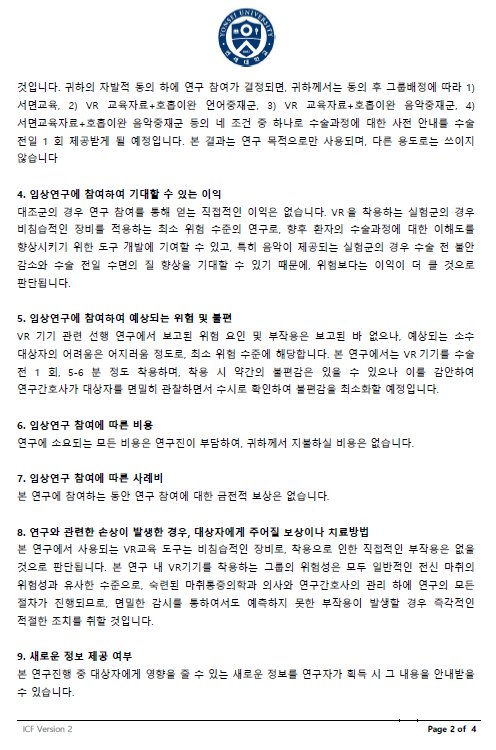

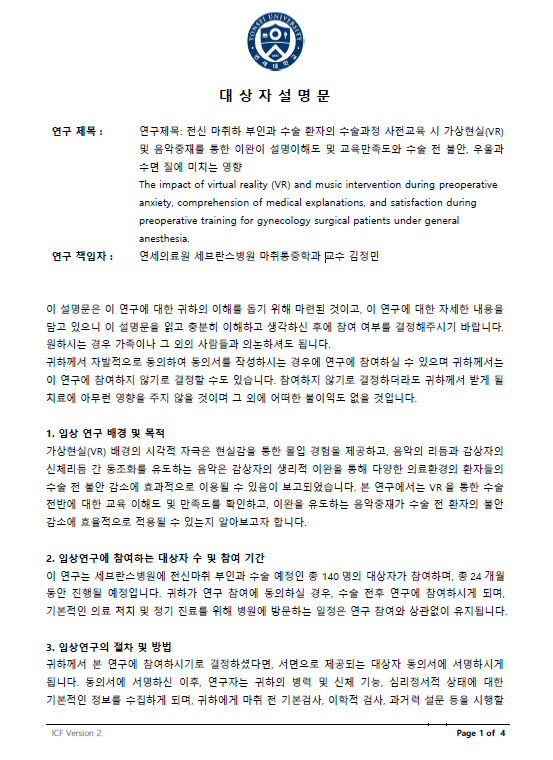
Sample Consent Form

#
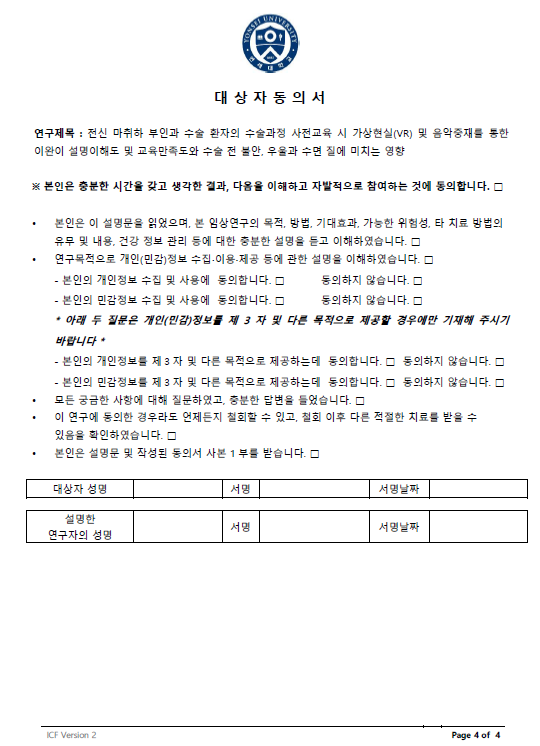

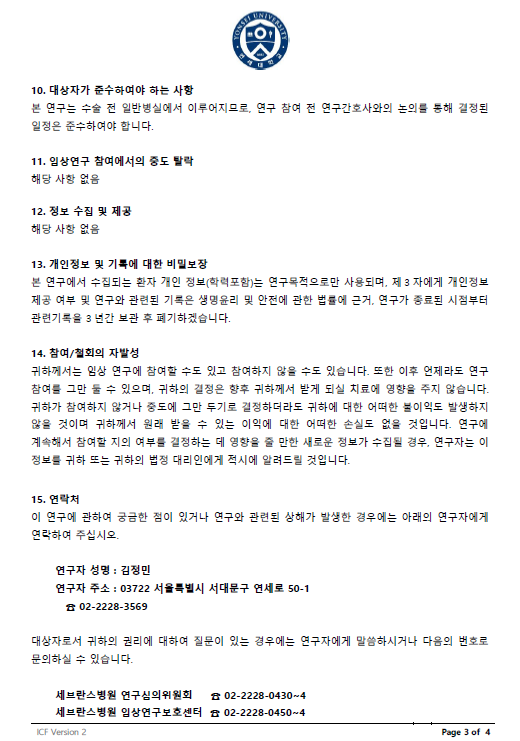


# -

# Educational materials (written, VR screenshots, music score)

Written form


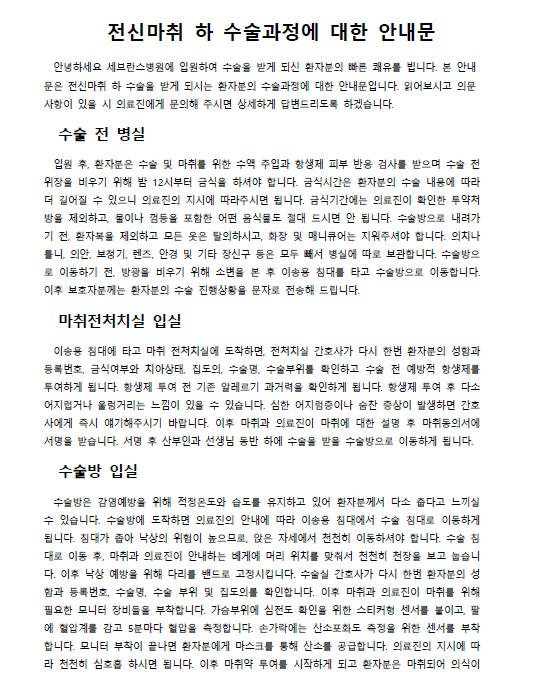


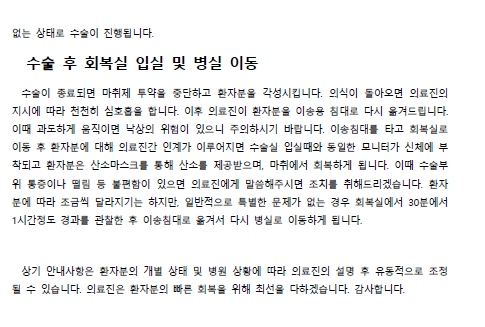


VR screenshots


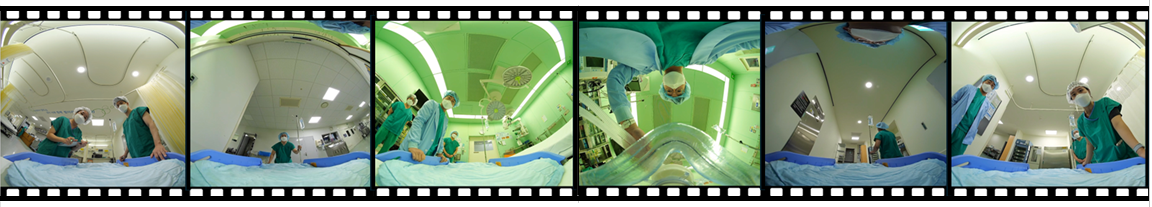


Music


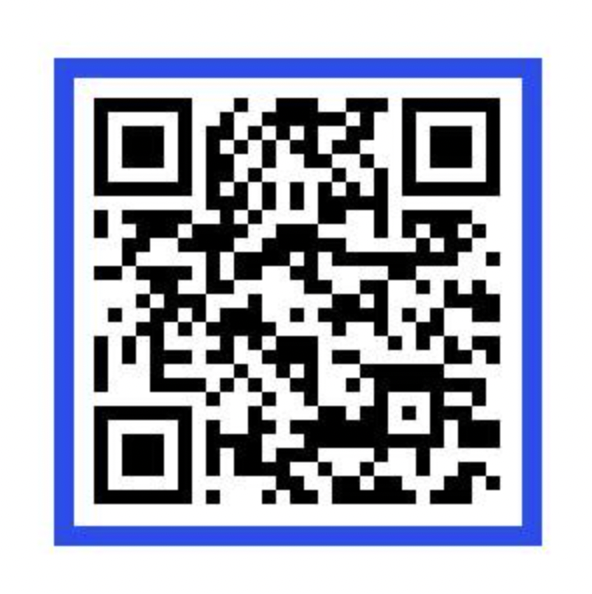

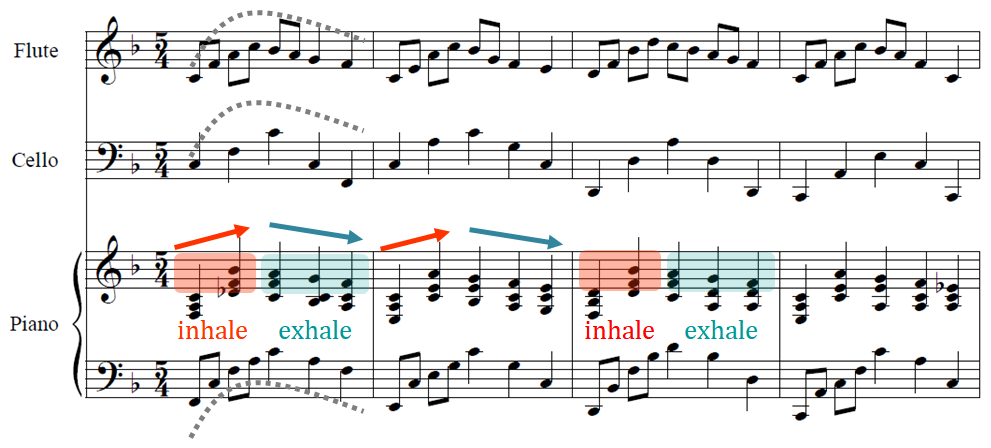

Supplement: Supplementary file 2 — Supplementary Material 2 [file 41598_2026_51676_MOESM2_ESM.docx]
